# Supplementary material for: Laugh before You Study: Does Watching Funny Videos before Study Facilitate Learning?
Source: Int J Environ Res Public Health. 2022 Apr 7;19(8):4434. doi: 10.3390/ijerph19084434 (PMC9030648; doi:10.3390/ijerph19084434)
Supplement: Supplementary file 1 [file ijerph-19-04434-s001.zip › ijerph-1636908-supplementary.pdf]

Knowledge pretest

1.True or false question. Tick √ for the right and tick √ for the wrong (1 point for each question, 3 points in total)

(1) Gold is not a mineral. (　　　　)

(2) The rocks are hard. (　　　　)

(3) Coal is a kind of rock. (　　　　)

2. Single choice. (1 point for each question, 2 points in total)

(1)Of the minerals that make up granite, the hardest is (　　　　)

A) mica　B) quartz　C) diamond

(2) What is true about the composition of rocks? (　　　　)

A) It is composed of one mineral　B) It is composed of three or more minerals　C)It is composed of one or more minerals

3. Multiple choice questions. (3 points for each question, 6 points in total)

(1) Of the following statements about rocks, which are true (　　　　)

A) Rocks must contain many minerals.

B) There are many kinds of rocks and many characteristics.

C) The particles that make up the rock may be coarse or fine, and the finer particles are smoother to the touch.

(2) The following characteristics of rocks are(　　　　)

A) color　B) length　C) gloss

Knowledge retention test

1. Fill in the blanks (1 point for each blank, 7 points in total)

(1) Granite is composed of__________________、__________________、__________________

(2) Of the three minerals in granite, the one that is transparent and glassy is__________________.

(3) There are __________________minerals have been discovered in the world.

(4) Minerals rarely exist alone in nature. They are usually mixed together to form__________________.

(5)______________________ is the hardest mineral found in nature.

2.True or false questions, tick √ for the right and tick √ for the wrong (1 point for each question, 3 points in total)

(1) Minerals are important materials that make up the rocks of the earth's crust. (　　　　)

(2) Granite is all the same color. (　　　　)

(3) Sand is a kind of rock. (　　　　)

3. Picture recognition. (Please write the name of the rock below the picture, 2 points for each

question, 8 points in total)

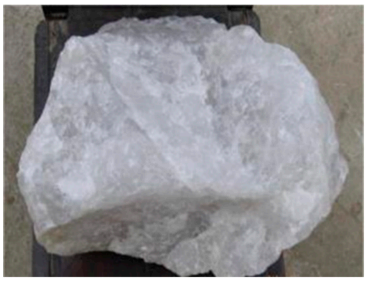
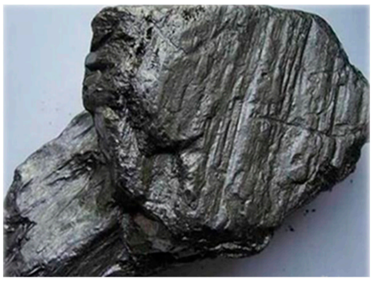

( ) ( )

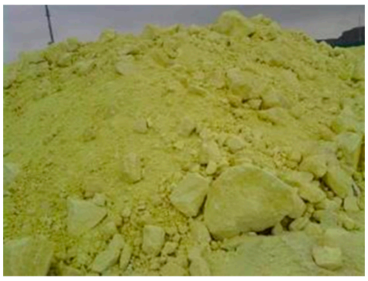
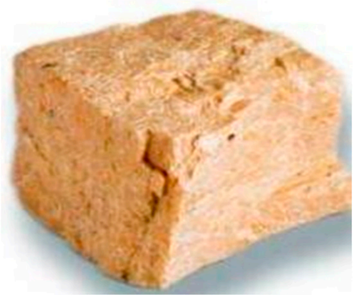

( ) ( )

Knowledge transfer test

I. True or false questions. Tick √ for the right and tick √ for the wrong (1 point for each question, 4 points in total)

(1) Rocks and minerals are natural. ( )

(2) Rocks and minerals are the mineral resources of the earth, not the resources that people produce and live in. ( )

(3) Pebbles contain minerals. ( )

(4)The more minerals a rock contains, the harder it is. ( )

2. Single choice questions (1 point for each question, 5 points in total)

(1) Illuminate the following objects with a flashlight. Which object is transparent ( )

A. quartz B. mica C. glass

(2) The reflection of light on the surface of rock forms the reflection of light on the surface of rock. ( )

A) color B) gloss C) transparency

(3) Of the following minerals, the one with the least hardness is ( )

A) quartz B)feldspar C) graphite

(4) Quartz, feldspar and mica, which are the hardest of the three minerals. ( )

A) quartz B) feldspar C) mica

(5) The luster of quartz, feldspar, mica and other minerals is close to ( )

A) metallic B) clay C) glass

3. Open questions (2 points for each example)

Name two examples of the use of rocks in your life.
